# P04-02 Are Older Adults Adhering to the Strength Component of the Physical Activity Guidelines?

**DOI:** 10.1093/eurpub/ckac095.056

**Published:** 2022-08-29

**Authors:** Ashley Gluchowski, Helena Bilsborough

**Affiliations:** University of Manchester, Manchester, United Kingdom

**Keywords:** Exercise, Strength, Training, Promotion, Policy

## Abstract

**Background:**

In 2019, the UK prominently placed the strength recommendations on their Chief Medical Officers' Physical Activity Guidelines infographic. The purpose of this study was to offer a nuanced description of older adults' awareness, understanding, and participation in activities that meet the strength component, as well as their perceived barriers to strength training participation.

**Methods:**

Older adults living in the UK (n = 15, 70±3.3 years) volunteered to participate in one 30-minute, semi-structured, one-on-one interview on Zoom with the lead author. Advertisements were placed in ageing charity newsletters. People who identified as 65 years old or over and living in the UK were asked to respond via email if interested.

**Results:**

Awareness. None of our participants were aware of the strength recommendations. “I honestly can't say that I ever recall seeing that.”

Understanding and Action. Walking was the most common modality for participants who believed they were meeting the strength guidelines. “I think I'm more than meeting them because … I do masses of walking …”

Suggestions for Improvement. Adding more detail to the guidelines and separating the guidelines based on ability, rather than chronological age, was suggested. “It's a bit subjective as to what counts as building strength.”

Barriers to Strength Training. Barriers included misconceptions about strength training in later life, “You know, you always know, don't overload yourself…I never push it.” and a lack of options for older adults who are not quite ready for classes for the oldest old, “There's a big cohort of us that are what you might call young old and the provision for us who are fit and active is sadly missing…the classes that are on are always for the older old”

**Conclusion:**

Our participants reported an unawareness of the strength guidelines. Adherence reporting to the strength guidelines should be interpreted with abundance of caution, as older adults are largely unaware of what activities fulfill this requirement. Researchers & practitioners can influence the many barriers to strength training participation primarily with dissemination of accurate information and providing age & ability-appropriate strength prescription.

